# 47 patients with *FLNA* associated periventricular nodular heterotopia

**DOI:** 10.1186/s13023-015-0331-9

**Published:** 2015-10-15

**Authors:** Max Lange, Burkhard Kasper, Axel Bohring, Frank Rutsch, Gerhard Kluger, Sabine Hoffjan, Stephanie Spranger, Anne Behnecke, Andreas Ferbert, Andreas Hahn, Barbara Oehl-Jaschkowitz, Luitgard Graul-Neumann, Katharina Diepold, Isolde Schreyer, Matthias K. Bernhard, Franziska Mueller, Ulrike Siebers-Renelt, Ana Beleza-Meireles, Goekhan Uyanik, Sandra Janssens, Eugen Boltshauser, Juergen Winkler, Gerhard Schuierer, Ute Hehr

**Affiliations:** Department of Neurosurgery, University of Regensburg, Medical Center, Franz-Josef-Strauss-Allee 11, 93053 Regensburg, Germany; Department of Neurology, Epilepsy Center, University of Erlangen, Medical Center, Erlangen, Germany; Institute of Human Genetics, University of Muenster, Muenster, Germany; Department of General Pediatrics, Muenster University Children’s Hospital, Muenster, Germany; Neuropädiatrie, Schön Klinik Vogtareuth, Vogtareuth, Germany und Paracelsus Medical University, Salzburg/Austria, Salzburg, Austria; Department of Human Genetics, Ruhr-University Bochum, Bochum, Germany; Praxis fuer Humangenetik, Klinikum Bremen-Mitte, Bremen, Germany; Institute of Human Genetics, Heidelberg University, Heidelberg, Germany; Klinik für Neurologie, Klinikum Kassel and Medical School, Kassel, Germany; Department of Neuropediatrics, University of Giessen, Giessen, Germany; Praxis fuer Humangenetik, Homburg, Saar Germany; Ambulantes Gesundheitszentrum der Charité (Humangenetik), Universitätsmedizin Berlin, Berlin, Germany; Department of Neuropediatrics, Klinikum Kassel, Kassel, Germany; Institut für Humangenetik, Uni Jena, Jena, Germany; Department of Pediatrics, University of Leipzig Medical Center, Leipzig, Germany; Center for Human Genetics, Regensburg, Germany; Genetics Clinic, Guy’s Hospital, Guy’s and St Thomas’ NHS Foundation Trust, London, United Kingdom; Zentrum für Medizinische Genetik, Hanusch-Krankenhaus der Wiener Gebietskrankenkasse, Wien, Austria; Centre for Medical Genetics, Ghent University Hospital, Ghent, Belgium; Division of Neuropediatrics, University Children’s Hospital Zürich, Zürich, Switzerland; Division of Molecular Neurology, University Hospital, Friedrich-Alexander-University Erlangen-Nuernberg, Erlangen, Germany; Department of Neuroradiology, University of Regensburg, Medical Center, Regensburg, Germany; Department of Human Genetics, University of Regensburg, Medical Center, Regensburg, Germany

**Keywords:** Periventricular nodular heterotopia, Filamin A, Imaging, Score, Seizures, Phenotype

## Abstract

**Background:**

Heterozygous loss of function mutations within the Filamin A gene in Xq28 are the most frequent cause of bilateral neuronal periventricular nodular heterotopia (PVNH). Most affected females are reported to initially present with difficult to treat seizures at variable age of onset. Psychomotor development and cognition may be normal or mildly to moderately impaired. Distinct associated extracerebral findings have been observed and may help to establish the diagnosis including patent ductus arteriosus Botalli, progressive dystrophic cardiac valve disease and aortic dissection, chronic obstructive lung disease or chronic constipation. Genotype-phenotype correlations could not yet be established.

**Methods:**

Sanger sequencing and MLPA was performed for a large cohort of 47 patients with Filamin A associated PVNH (age range 1 to 65 years). For 34 patients more detailed clinical information was available from a structured questionnaire and medical charts on family history, development, epileptologic findings, neurological examination, cognition and associated clinical findings. Available detailed cerebral MR imaging was assessed for 20 patients.

**Results:**

Thirty-nine different FLNA mutations were observed, they are mainly truncating (37/39) and distributed throughout the entire coding region. No obvious correlation between the number and extend of PVNH and the severity of the individual clinical manifestation was observed. 10 of the mutation carriers so far are without seizures at a median age of 19.7 years. 22 of 24 patients with available educational data were able to attend regular school and obtain professional education according to age.

**Conclusions:**

We report the clinical and mutation spectrum as well as MR imaging for a large cohort of 47 patients with Filamin A associated PVNH including two adult males. Our data are reassuring in regard to psychomotor and cognitive development, which is within normal range for the majority of patients. However, a concerning median diagnostic latency of 17 to 20 years was noted between seizure onset and the genetic diagnosis, intensely delaying appropriate medical surveillance for potentially life threatening cardiovascular complications as well as genetic risk assessment and counseling prior to family planning for this X-linked dominant inherited disorder with high perinatal lethality in hemizygous males.

## Background

Neuronal heterotopia (NH) is one of the most frequent malformations of cortical development observed in patients with epilepsy [1]. It is characterized by an ectopic accumulation of neurons that fail to migrate to the cerebral cortex [2]. NH is a morphologically and etiologically heterogeneous condition, in particular solitary nodules may be observed as an unspecific finding either isolated or with various complex brain malformations. The most frequent symmetric manifestation of periventricular nodular heterotopia (PVNH) is located along the walls of both lateral ventricles predominantly in females and results from heterozygous *loss of function* mutations in the X-linked *FLNA* gene [3, 4]. It is associated with high intrauterine and perinatal lethality in hemizygous males presumably from excessive bleeding, however on rare occasions boys and adult hemizygous male carriers of *FLNA* mutations have been reported [4, 5]. The gene product Filamin A is a large cytoplasmic actin-binding and cross-linking protein of diverse functions including initiation of cell migration and spreading, coagulation and aspects of vessel wall integrity [6–8]. Cellular function of Filamin A is further modulated by dimerization with the homologous protein Filamin B, which may rescue defective Filamin A depending upon the cellular environment [6]. Functional imaging indicates that the *FLNA* associated ectopic cortical neurons are functionally integrated into motor circuits [9]. The phenotype in females with heterozygous *FLNA* loss of function mutation is very variable. Difficult to treat epileptic seizures are the core clinical finding in about 90 % of the patients and may only start in adulthood [3, 8, 10]. Additional neurological findings are rather discrete and may include deficits in reading, processing speed and executive functions, only detectable in subtle neurocognitive testing in about 80 % of patients [11]. Penetrance in heterozygous *FLNA* mutation carriers is reduced and asymptomatic PVNH may be detected through predictive carrier testing or incidentally in cerebral MR imaging as the only manifestation of a *FLNA* mutation.

Other rare genetic causes of PVNH include chromosomal imbalances and submicroscopic genomic copy number variations (CNV) and rare mutations within the *ARFGEF2* gene resulting in autosomal recessive inherited PVNH. *ARFGEF2* encodes the brefeldin-inhibited guanine exchange factor 2 (BIG2) protein [12]. BIG2 plays a key role in vesicle transport between the trans-Golgi apparatus und the cell membrane. These children are more severely affected and present with early onset epilepsy, congenital microcephaly, severe mental retardation and increased susceptibility to infections [13].

Cerebral MR imaging of *FLNA*-associated PVNH in addition often reveals an enlarged pericerebellar cerebrospinal fluid space in the presence of normal cerebellar and 4th ventricle anatomy. This will be referred to as “mega cisterna magna” [14].

Less frequent *FLNA*-associated extracerebral manifestations include persistent ductus arteriosus Botalli in the newborn, chronic obstipation, an Ehlers-Danlos-like phenotype affecting connective tissues, cardiac valve disease as well as chronic obstructive lung disease and OPD spectrum skeletal phenotypes (oto-palato-digital syndrome) [15–20].

Small series of PVNH patients on electrophysiological, radiological, histological findings and neurosurgical outcome after epilepsy-surgery have been reported without genetic data [1, 16]. Here we describe for the first time the phenotypic spectrum of a genetically defined larger patient cohort with *FLNA* mutations including neuroimaging, neurological and extracerebral findings.

## Patients and methods

### Patients

Samples of all patients were referred for genetic testing of the *FLNA* gene by neuropediatricians, neurologists or geneticists with informed consent of the patients and/or their parents. For a subset of 34 patients clinical and anamnestic data were collected retrospectively using a structured, standardized questionnaire specifically designed for patients with *FLNA*-associated PVNH or from clinical charts, respectively (Table 1). The questionnaires systematically asked for family history/miscarriages, epilepsy/seizures, clinical findings on neurological examination, cognitive development and developmental milestones as well as “associated clinical findings” (ACF) as listed in Table 2. The questionnaires were processed by the patients themselves together with their physicians and/or caregivers. In 20 patients original MR images were available for assessment. Clinical data from these questionnaires as well as individual clinical charts from the remaining patients were evaluated as available and correlated with the obtained genetic results and findings of MR imaging.

**Table 1:** Summary of the clinical and genetic data

Family/pat		Age at genetic diagnosis (years)	Clinical subgroup	Associated clinical findings (ACF)	cDNAsequencealteration	Proteinchange	Mutation type
1		16,9	4	2	c.120delG heterozygous	p.Trp41GlyfsX17	Frameshift
2		34,3	3		c.289C>T heterozygous *de novo* (both parents wildtype)	p.Pro97Ser	Missense
3		22,1			c.464G>A heterozygous *de novo*	p.Trp155^*^	Nonsense
4		31,8	2	0	c.961G>T heterozygous	p.Glu321^*^	Nonsense
5		37,6	4		c.1065+1G>C heterozygous	IVS7+1G>C ds	Splice site mutation
6		25,4	3	1	c.1065+1G>T heterozygous	IVS7+1G>T ds	Splice site mutation
7		17,1	4	1	c.1087C>T heterozygous	p.Gln363^*^	Nonsense
8		41,4	2	0	c.1351_1352insAG heterozygous	p.Gly452ArgfsX47	Frameshift
9		32,1			c.1580_1581insCAGAAGGACCTGGGGGATG heterozygous	p.Arg527ProfsX100	Frameshift
10		57,0			c.2022+1G>A heterozygous	IVS13+1G>A ds	Splice site mutation
11	II/1	3,4	4	0	c.2192dupA heterozygous maternal	p.Tyr731^*^	Nonsense
	I/1	31,9	3	0	c.2192dupA heterozygous *de novo*	p.Tyr731^*^	Nonsense
12		30,0	2	1	c.2565+2T>G heterozygous	IVS17+2T>G ds	Splice site mutation
13		19,0			c.2612dupA heterozygous *de novo*	p.Asp871GlufsX4	Frameshift
14		17,9	1	1	c.2943_2944_ + 2delGAGT heterozygous *de novo*	IVS20_-2_ + 2del ds	Splice site mutation
15	II/1	33,3			c.2983_2987delTCAAA heterozygous maternal	p.Ser995GlyfsX31	Frameshift
	I/1	55,4			c.2983_2987delTCAAA heterozygous	p.Ser995GlyfsX31	Frameshift
16		17,2	2	2	c.3174delT heterozygous	p.Leu1059TrpfsX12	Frameshift
17		16,6	2	1	c.3742C>T heterozygous	p.Gln1248^*^	Nonsense
18		37,3	3	1	c.4294C>T heterozygous	p.Gln1432^*^	Nonsense
19		51,7	3	0	Deletion exon 25 heterozygous	?	Exondeletion
20		1,7			c.4303+2T>G heterozygous	IVS25+2T>G ds	Splice site mutation
21		18,0			c.4576G>T heterozygous	p.Gly1526^*^	Nonsense
22		12,4			c.4720delG heterozygous *de novo*	p.Asp1574ThrfsX39	Frameshift
23	II/1	33,0	2	0	c.4994dupA heterozygous maternal	p.Ile1666AspfsX12	Frameshift
	I/1	65,8			c.4994dupA heterozygous	p.Ile1666AspfsX12	Frameshift
24^a^	II/1^a^	30,7	1	0	c.5686G>A heterozygous paternal	p.Gly1896Arg/Splice	Splice site mutation
	II/2^a^	28,9	3	1	c.5686G>A heterozygous paternal	p.Gly1896Arg/Splice	Splice site mutation
	I/1 male^a^	62,2	2	0	c.5686G>A mosaicism	p.Gly1896Arg/Splice	Splicesitemutation
25		47,0	2	1	c.6321C>A heterozygous	p.Cys2107^*^	Nonsense
26		17,8			c.6898C>T heterozygous	p.Gln2300^*^	Nonsense
27		24,9	1	2	c.6908-2A>G heterozygous	IVS42-2A>G	Splice site mutation
28		2,5	4	2	c.6994_7003dupGCCCGCCGCC heterozygous	p.Leu2335ArgfsX8	Frameshift
29	Male	38,2	2	3	c.7055_7070delCTTTTGCAGTCAGCCT mosaicism	p.Ser2352^*^	Nonsense
30		44,0	2	2	c.7075_7077delinsT heterozygous	p.Gly2359CysfsX25	Frameshift
31		21,7			c.7115C>G heterozygous	p.Ser2372^*^	Nonsense
32	II/1	24,5	2	0	Heterozygous deletion exon 44 maternal	Loss of function	Exon deletion
	II/2	24,1	2	0	Heterozygous deletion exon 44 maternal	Loss of function	Exon deletion
	I/1	53,7	4	0	Heterozygous deletion exon 44	Loss of function	Exon deletion
33		27,9			c.7223delG heterozygous *de novo*	p.Gly2408AlafsX45	Frameshift
34		1,0	4	2	c.7255C>T heterozygous	p.Arg2419^*^ *de novo*	Nonsense
35		42,2	2	0	c.7533delC heterozygous	p.Phe2512SerfsX24	Frameshift
36		7,9	4	1	c.7714G>A heterozygous; unclassified variant	p.Val2572Ile - VUS3	Missense/splice site mutation?
37		8,2	1	8	c.7840dupT heterozygous	p.Tyr2614LeufsX136	Frameshift
38	II/1	16,5	4	1	Heterozygous deletion exons 40 to 3′UTR maternal	Loss of function	Exon deletion
	I/1	41,5	4	0	Heterozygous deletion exons 40 to 3′UTR *de novo*	Loss of function	Exon deletion
39		31,3	3	2	Deletion exon 46 + duplication exons 4–22 *de novo* (both parents wildtype)	Loss of function	Complex genomic rearrangement

The two male patients are separately indicated in the family/patients row

^a^family 24 has previously been published including detailed clinical data [5] and surgical correction of gastrointestinal dysfunction of patient 34 in [23]

**Table 2:** Associated clinical findings (ACF) observed in 34 patients from our cohort

	n observed per group vs. total percentage				
Cardiovascular	I	II	III	IV	Total (%)
Aortic valve insufficiency	1	4		3	8 (23.5)
Aortic dilation	1				1 (2.9)
Mitral valve insufficiency				1	1 (2.9)
Persistent ductus arteriosus	2	1	2	1	6 (17.6)
Cerebral aneurysm	1		1		2 (5.9)
Internal findings					
Gastrointestinal dysfunction	1			1	2 (5.9)
Liability to hematoma	1		1		2 (5.9)
Obstructive lung disease		2			2 (5.9)
Dysmorphism/physical handicaps					
Joint hypermobility	2	1		2	5 (14.7)
Musclular hypotonia	1				1 (2.9)
Talipes				1	1 (2.9)
Skin hyperextensible	1	1			2 (5.9)
Craniofacial dysmorphism	1	1		1	3 (8.8)

Associated clinical findings (ACFs) were documented in 19 of the patients (55.9 %)

However, in absolute numbers there were 12 ACFs in 4 patients in group I (average 3 per patient), 10 in 13 patients in group II (0.77/patient), 4 in 7 patients in group III (0.43/patient) and 10 in 10 patients in group IV (1,0/patient). The load of multiple different ACFs seems to be higher in younger patients. Patent ductus arteriosus Botalli (PDA) in 5 patients and cardiac valve disease in 8 patients were the most frequently observed ACFs, they showed no correlation to the patients’s age or neurological phenotype

### Mutation analysis

Genomic DNA was prepared from peripheral blood. With informed consent the entire *FLNA* coding sequence and flanking splice sites (reference sequence NM_001110556.1) were amplified by PCR and analyzed for potential sequence variations by direct sequencing of PCR products using an ABI Prism Big-Dye Terminator Cycle Sequencing Kit version 1.1 and ABI 3100 DX Avant sequencer; Applied Biosystems, Foster City, Calif., USA). Reaction protocols have previously been described [5, 15, 16]. Larger *FLNA* exon deletions or duplications were identified using the commercially available multiplex ligation dependent probe amplification (MLPA) kit P-061 (MRC-Holland, Amsterdam, the Netherlands).

## Results

### Mutation analysis

**Fig. 1:**
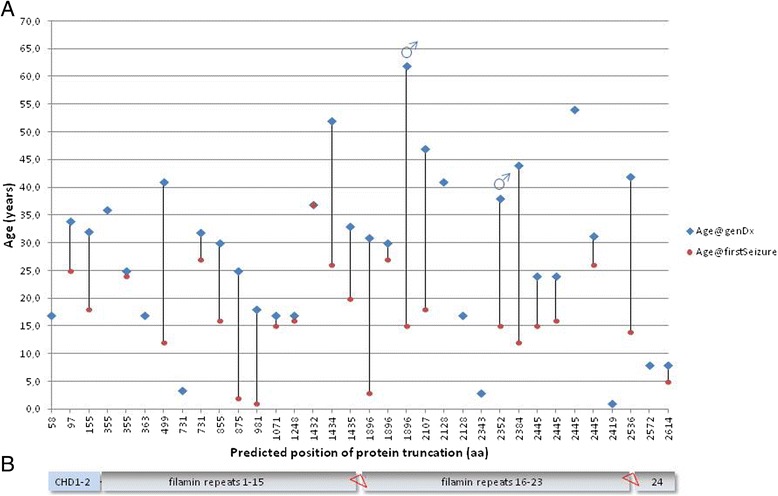
**a** Patient age at onset of the seizure disorder (*red*) and at genetic diagnosis (*blue*) with diagnostic latency (*black line*); **b** schematic structure of the corresponding Filamin A protein (*not to scale*) with the two NH2-terminal actin binding calponin homology domains (CHD1-2) and 24 immunoglobulin-like filamin repeat domains interrupted by two hinge regions (*red triangles*)

The patient cohort consists of 2 male and 45 female patients from 39 independent families with a median age of 28.5 years at the time of genetic diagnosis (Table 1, Fig. 1). For 14 families maternal samples were available with additional paternal samples for 3 of them (families 2, 24 and 39) and results of parental carrier testing compatible with a *de novo* mutation for 10 index patients.

For 5 of the remaining index patients the carrier status of their mothers or daughter (family 11) was only recognized during family studies at an age between 16 and 65 years (median 44,1 years), where none of those carrier relatives so far had experienced seizures or other clinical abnormalities previously associated with *FLNA* mutations.

Identified *FLNA* mutations are distributed throughout the entire *FLNA* gene and include 37 truncating mutations (frameshift: 13, nonsense: 12, splice site: 8; exon deletions: 4). Missense variants were only identified in 2 patients. For one of these two missense variants p.Pro97Ser (c.289C>T) the absence in both parental samples further suggests its functional and clinical relevance as *de novo* mutation. It has not been annotated in LOVD3, public HGMD or Ensemble or in other published patients or controls available to us. In close vicinity additional pathogenic missense mutations p.Glu82Val and p.Met102Val have been described previously [21, 22]. For the other patient with the missense variant p.Val2572Ile no parental samples were available. It has been annotated as rs377518545 and observed in heterozygous state in 2 out of 2332 females and none of the 1784 males from the ESP6500 cohort of European Americans, nor in any of the 3358 ESP6500 alleles of African American origin. Bioinformatic assessment suggested the activation of a cryptic splice site. However, in the absence of parental samples and clear evidence for functional and clinical relevance we currently consider this as an unclassified variant.

**Fig. 2:**
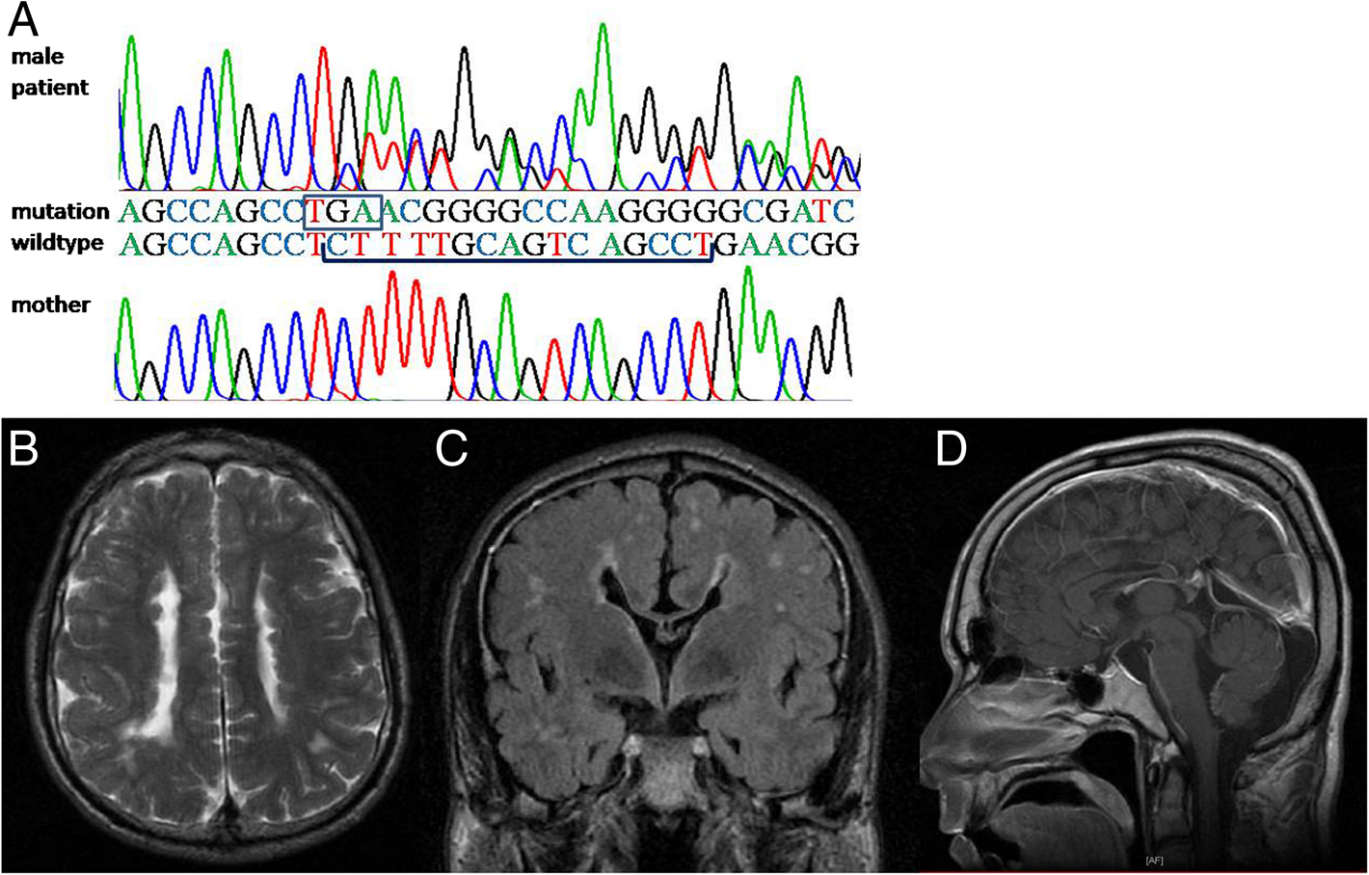
**a** High grade mosaic *FLNA* mutation c.7055_7070delCTTTTGCAGTCAGCCT in peripheral blood (*upper sequence*) of a 36 year old male patient 29 with normal male karyotype 46,XY, resulting in the nonsense mutation p.Ser2352*, novel stop codon boxed. Wildtype sequence of the healthy mother below. **b**–**d** cerebral MR imaging demonstrating extended PVNH bilaterally (**b** and **c**), diffuse white matter abnormalities and inward rotated anterior ventricular horns (**c**), hypoplastic corpus callosum and large mega cisterna magna (**d**)

For one adult male patient in this study (family 29) a 16 bp deletion in exon 44 was observed in high grade mosaic state in the available blood sample with introduction of a stop codon directly at the mutation site (p.Ser2352*; Fig. 2a). Based on the available chromatograms of the Sanger sequence at least 60 % of the FLNA alleles in genomic DNA prepared from peripheral blood were estimated to carry the 16 bp deletion. A normal male karyotype 46,XY in lymphocytes from peripheral blood was confirmed and in addition fluorescence *in situ* hybridization among 100 further metaphases and interphase nuclei did not reveal any cell with more than one signal for the X chromosome, thus excluding clinically relevant X gonosomal mosaicism for 47,XXY as an alternative explanation. His mutation was not present in a blood sample of his mother, thus further confirming *de novo* occurrence of this nonsense mutation during early embryonal development.

The only other male patient of this study (# I/1 from family 24) was also found to carry a potentially truncating *FLNA* splice site mutation c.5686G>A in mosaic state estimated to affect 40 to 45 % of FLNA alleles in peripheral blood, and was previously published [5].

### Clinical findings

For 34 patients detailed clinical data were available (mean age at genetic diagnosis: 28.5 years; range: 1 – 54 years): data from standardized clinical questionnaires were assessed from 24 patients. For further 10 patients clinical data from medical records could be evaluated.

According to the age at first seizure patients were divided into 4 clinical subgroups (Table 1):

#### Subgroup 1 seizure-onset in childhood

**Fig. 3:**
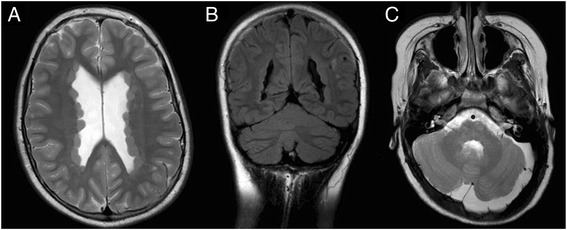
**a-c** cMR imaging of 11 year old female patient 37 with complex *FLNA* associated phenotype including severe connective tissue disorder with gastrointestinal, cardiac and vascular manifestation, resulting from heterozygous C-terminal *FLNA* frameshift mutation c.7840dupT. **a** and **b** extended cortexisodense PVNH bilaterally; (**b**) white matter abnormalities and (**c**) large mega cisterna magna

Four female patients were younger than 11 years at the time of their first seizure (mean age 2.8 years; age range: 1–5 years). The causal *FLNA* mutation in this subgroup was identified with a mean latency of 17.8 years (range 3–28, Fig. 1). Three of these patients carried heterozygous splice site mutations (patients # 14, 27 and II/1 from family 24), for which specific consequences on the abnormal *FLNA* transcript and protein cannot be predicted. The fourth of these female patients (# 37) is heterozygous for the most C-terminal truncating mutation identified in our cohort with abrogation of the regular stop codon, elongated transcript and most severe complex phenotype including severe and multi-organ connective tissue manifestation in the presence of moderate PVNH (Fig. 3).

#### Subgroup 2 seizure-onset during adolescence

In 13 clinically documented patients the first seizure occurred during adolescence at a mean age of 15.5 years (range 12–20). Diagnostic latency regarding mutation detection in this subgroup was 18.3 years (range 1–47, Fig. 1). Two of these patients were males with mosaic truncating mutation detected in peripheral blood [5].

#### Subgroup 3 seizure-onset in adulthood

Only 7 of the clinically documented patients had their first seizure beyond the age of 20 years (mean: 27.4; range: 24–37) with an average latency for genetic diagnosis of 7.2 years (range 0–26).

#### Subgroup 4 no seizures

The fourth subgroup of 10 females had not experienced any seizures at the time of genetic diagnosis (mean age at genetic diagnosis: 19.7 years (range: 1–54 years). All of these patients were neurologically normal, reasons for extended neurological examination including MR imaging and EEG were either unexplained recurrent headache/migraine (3 patients) or other clinical features (5 patients) and/or predictive carrier testing for a familial *FLNA* mutation (3 patients).

### Psychomotor development and cognition

Early psychomotor milestones data were available for 21 patients. In 18 patients they were reached within normal limits. A selectively retarded speech development was reported for one patient from subgroup 2 (patient # 4). A more pronounced early developmental delay has been observed in two female patients of subgroup 1 (patient # II/1 from family 24 and patient # 37) with a more complex phenotype including connective tissue involvement. Cognitive impairment was noted in 4 patients - predominantly with seizure onset prior to the age of 20 years: in subgroup 1 in two of four patients (50 %) and only one patient each in subgroups 2 (7.7 %) and 4 (10.0 %). Moreover, educational data were available for 24 patients: all but two of them were able to attend regular school and obtain professional education according to age. A more profound cognitive impairment is documented for only one female index patient (patient # II/1 from family 24) [5].

### Neurological findings

The clinical neurological examination was normal in 33 of 34 patients, in only one patient from subgroup 1 (patient # 37) with a more complex phenotype muscular hypotonia has been documented. 5 patients from 4 families reported recurrent headache or migraine.

### Neuroimaging findings

MR imaging confirmed bilateral nodularly arranged, heterotopic gray matter with periventricular distribution in all assessed patients. The extent of PVNH greatly differed amongst patients, as did the number of associated imaging findings (AIF, Table 3). 14 patients showed extensive, bilateral, confluent heterotopic conglomerates of neuronal tissue (Figs. 1b and 2a, b).

**Table 3:** Neuroimaging findings

Associated imaging findings (AIF):	Secondary imaging findings:
- Mega cisterna magna (14/15)	- Dilated ventricles (2/15)
- Deformation anterior horns lateral ventricles (8/12)	- “Cortical thinning” (0/12)
- White matter lesions (7/12)	- Intracranial aneurysms (0/12)
- Corpus callosum hypoplasia (6/15)	
- Abnormal cortical gyration (1/12)	

A mega cisterna magna was observed in 18 of 20 patients with available sagittal MR imaging (Figs. 1d, 2c and 3c). More importantly, in 9 of 20 patients we noted specifically deformed anterior horns of the lateral ventricles with characteristic inward rotation (Figs. 1c and 3b), which have not been described before by other groups.

**Fig. 4:**
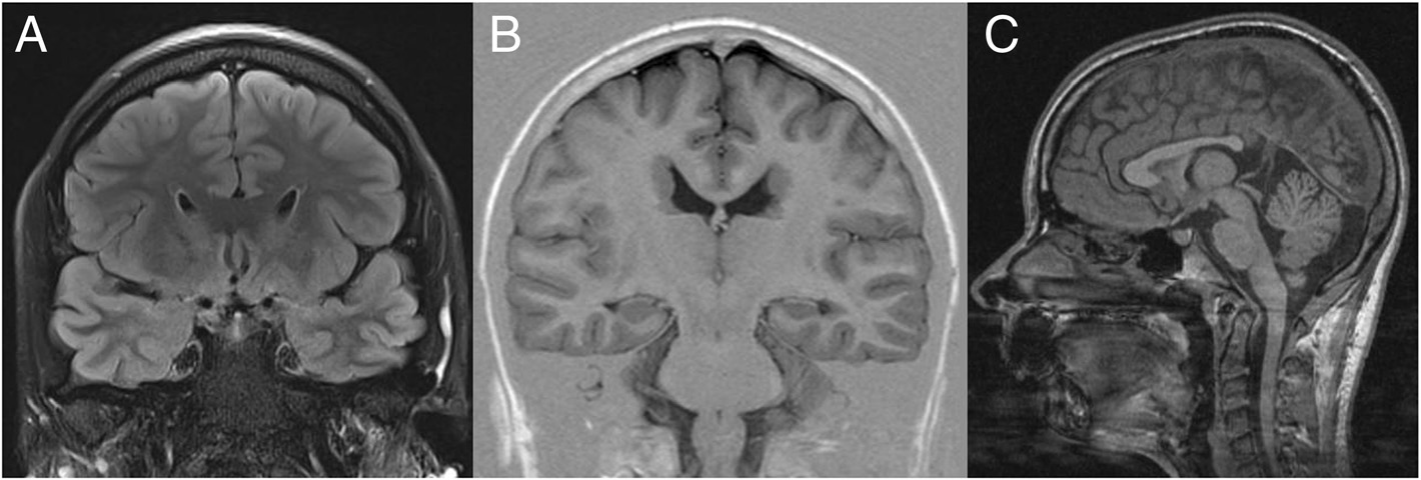
**a**, **b** coronal cMR imaging of the anterior ventricular horns with normal configuration (**a**) in 27 year old female patient 39 with PVNH only along the central and occipital parts of the lateral ventricular walls and (**b**) with the more frequently observed anterior heterotopia and abnormal inward rotation in 16 year old patient 16. **c** Sagittal cMR imaging of 17 year old patient 26 with characteristically shortened hypoplastic corpus callosum and large mega cisterna magna

Corpus callosum hypoplasia was recognized in 8 of 20 patients with predominant thinning of the splenium (Figs. 2d and 4c). For none of the available MR scans convincing dilated ventricles or hydrocephalus were observed. However, focal anomalies of the cortical gyral pattern could be seen in one patient (index patient from family 5 with unilateral focal occipital cortical dysplasia) and white matter lesions in 9 of 20 patients (Figs. 2c and 3b). Those white matter lesions were located subcortically in 8 patients (average age at genetic diagnosis: 26.4 years) and periventricularly in only one 62 year-old patient and in this instance may rather represent age-associated incidental findings.

In our cohort we did not observe a correlation between the extent of PVNH with the age of onset of seizures or overall clinical severity.

### EEG findings

EEGs results were available from 20 patients, 15 had epilepsy, 7 of those exhibited discharges in the scalp EEG, in 7 the EEG was normal, in 1 it showed focal slowing as the key finding.

In 5 patients from subgroup 4 (without epilepsy) with available EEG there were either unspecific changes (*n* = 3; average age 12.3 years), epileptic discharges (*n* = 1; EEG at the age of 8 years) or normal recordings (*n* = 1, age 3.4) were documented. MR scans were available for two patients with focal slowing from this subgroup 4 and confirmed moderate and severe PVNH, respectively as well as severe PVNH in one patient with normal EEG.

In subgroup 1, 2 of 3 EEGs were normal whereas the third EEG showed epileptic discharges. MRIs were available for 2 clinically severely affected patients of subgroup 1. One had a normal EEG despite of a seizure-onset at the age of 5: the MRI revealed a more severe cerebral phenotype (patient # 37; Fig. 3). The other had epileptic discharges in the EEG (patient # II/1, family 24).

In sugroup 2, three of 8 EEGs were normal, 4 showed epileptic activity and one was unspecifically disturbed (focal slowing). MRIs were obtainable for 6 patients: 3 with discharges in the EEG and 3 with a normal EEG. In subgroup 3, two of four EEGs revealed discharges and 2 were normal. Two MRIs were available: one patient with discharges and one with a normal EEG both had moderate PVNH.

### Pregnancy outcome

One or more miscarriages were reported for 6 of 39 families. In family 32 the mother of two affected twin sisters, all with heterozygous deletion of exon 44, reported 3 additional pregnancies with miscarriage around the 12th gestational week, her mother also had one miscarriage and 3 further live births (one boy and 2 girls). Evaluation of available questionnaires revealed 18 live born siblings of mutation carriers without obvious gender distortion (8 males, 10 females). However, for most of their mothers mutation status was unknown. No instance of a late miscarriage, stillbirth or induced abortion due to fetal malformations was documented in our cohort.

### Dysmorphic findings and connective tissue manifestation

We specifically asked for dysmorphic features, cardiac disease, skin and joints abnormalities as well as cerebral artery aneurysm and stenosis (Table 2). Cerebral arerial findings were present in two girls.

Patient # 34 presented with complete nonrotation of the bowel, absence of gastrophrenic ligaments, and nonfixation of the intestines requiring acute extensive abdominal surgery due to a wandering spleen with torsion and dislocation into the umbilical area associated with an upside-down volvulus of the stomach at the age of 15 months [23].

The same patient developed progressive dilatation of the aorta with a diameter of 33 mm at the level of the aortic root and 27 mm at the level of the ascending aorta with aortic regurgitation. At the age of 12 years the aortic valve and the ascending aorta were replaced with a 21 mm composite graft and a Saint Jude Medical aortic valve prosthesis. Additionally, cerebral Doppler studies revealed turbulent flow in the internal cerebral arteries bilaterally at the age of 7 years, MR angiography confirmed bilateral stenosis of the intradural internal cerebral arteries. The patient was placed on low dose aspirin, but the stenosis showed a slight progression over 4 years.

Patient #37 presented with extreme intestinal pseudoobstruction leading to severe constipation in adolescence [24]. Because of absent bowel movements despite the use of laxative and retrograde enemas, a Malone button was placed in the coecum for anterograde flushes at the age of 11 years. Constipation relapsed, and at the age of 14 years an ileostomy stoma was placed and relieved the constipation. Biopsies of the ascending colon and the terminal ileum revealed a relatively strong tunica muscularis mucosae and small focal lesions of fibrosis in the submucosa of the colon with normal ganglia cells in the myenteric plexus. In the same patient, a small aneurysm of 1 mm diameter of the anterior communicating artery was noted on MR angiography with a 3 Tesla high resolution MRI scanner at the age of 12 years and did not show progression on subsequent control imaging over 3 years. For 5 patients joint laxity or hypermobility was documented. Talipes were documented in patient # 36.

Dysmorphism was reported for 3 of 34 patients (8.8 %) including retrognathia, hypertelorism or low-set ears and one male patient (# 29) with more severe clinical manifestation within the OPD spectrum including the characteristic broad and flattened OPD I-like endphalanges of both feet.

Twelve of 34 patients were reported to be compromised by cardiovascular disease with documented persistent ductus arteriosus Botalli in 5 patients. Functionally relevant aortic valve insufficiency was observed in 8 patients with additional mitral valve insufficiency grade I in patient # 28 and progressive aortic dilatation in patient # 37.

The male patient # 29 developed a progressive obstructive lung disease with severely reduced vital capacity of 1.76 l and a FEV_1_ of 0.88 l at the age of 38 years, not allowing any physical exercise anymore and ultimately considering lung transplantation.

## Discussion

We present clinical, neuroimaging and mutation data of a large cohort of 47 patients with periventricular nodular heterotopia resulting from heterozygous or rare instances of mosaic *FLNA* mutations in males [16]. Our data confirm a specific *FLNA*-associated PVNH phenotype on MR imaging with bilateral, predominantly confluent nodular heterotopias extending along the entire length of the lateral walls of both lateral ventricles. Within our PVNH cohort we did not observe a correlation between PVNH extent and seizure phenotype or overall clinical course. Extensive and confluent PVNH in our cohort was not uncommon in asymptomatic *FLNA* mutation carriers or those from subgroup 3 with normal development and onset of seizures only in adulthood. Further functional studies may allow new insights into important protein domains and interaction partners critically contributing to the diverse effects of distinct *FLNA* mutations [25].

*FLNA*-associated PVNH is X-linked dominant inherited with a high risk of 50 % for male offspring to be severely affected. Interestingly, in our cohort no instances of stillbirths or late abortion were recorded, nor were milder affected male siblings with clinical findings of the *FLNA* phenotypic spectrum. This further strengthens the previously proposed hypothesis that (I) most hemizygous truncating *FLNA* mutations lead to predominantly early abrogation of intrauterine development [22], as might have been the case in the 3 miscarriages around the 12th week of gestation of patient # I/1 from family 32. (II) Our data from this large cohort further confirm, that *FLNA*-associated PVNH in liveborn males is rare and may only be compatible with postnatal development in the presence of critical amounts of correct functional full length cDNA either due to hypomorphic alleles, incomplete splice effects or somatic mosaicism for truncating mutations functionally similar to the situation in heterozygous female mutation carriers [4].

Early mutation detection is critical as it allows genetic counseling for women with heterozygous truncating *FLNA* mutations and her relatives prior to family planning, to non-directly discuss the mode of inheritance, clinical course and the options of prenatal genetic testing or even preimplantation genetic diagnosis in order to significantly support informed decisions. However, in 19 patients of our cohort the genetic diagnosis was established more than 10 years, for 11 of these even more than 20 years after seizure onset, although MR imaging with characteristic PVNH for some of them has been obtained for several years.

Furthermore, early recognition of mutation carriers is also important for extended medical surveillance [19, 20]: in our cohort cardiac disease was recorded for almost half of all patients. Persistent ductus arteriosus is commonly recognized in time and properly treated. However, dysplastic cardiac valve disease and less frequently aortic dilatation may be asymptomatic but still progress, while patients could benefit from early recognition, surveillance and appropriate treatment. The high proportion of cardiovascular manifestations in our cohort clearly underscores the importance of early and repeated cardiovascular surveillance, even in currently asymptomatic mutation carriers.

More recently, progressive obstructive lung disease has been documented as a rare clinical manifestation in *FLNA* mutation carriers [17]. In our cohort we present another male patient with mosaic truncating mutation and severe impairment of pulmonary function within his 4^th^ decade. Medical workup should therefore also include attention for clinical signs of impaired respiratory function.

While the predominant clinical manifestation is seizures [4, 11], which in more than half of the *FLNA* mutation carriers of our cohort developed in childhood or adolescence other less common manifestations were seen in our cohort including one patient each with with talipes deformation of the feet or muscular hypotonia. Our data further confirm the rare association of *FLNA* mutations with serious pseudoobstruction, initially reported by our group [20] and add the unusual finding of a complex intestinal malformation and malrotation as potentially more severe congenital gastrointestinal manifestation, requiring extensive abdominal surgery in those 2 patients during childhood [23]. In concordance with earlier reports laxity of joints and skin was rather frequent and also obstructive lung disease was seen as described above.

We specifically assessed psychomotor and cognitive development, which was normal for almost all PVNH patients in our cohort. Almost all patients were reported to be developed according to age, both regarding their psychomotor and cognitive skills. Our data are especially reassuring, as almost all patients could obtain and complete normal school and professional education according to age. Significantly delayed early development appears to be not common and was only noted in two patients from subgroup 1 with more complex and severe phenotype, requiring more intensive education in a school for children with special needs. In one of these girls, recurrent hospitalization with subsequent long and repeated miss-out times at school may be an important cofactor preventing attendance of a regular school.

## Conclusions

In summary, we here present genetic, clinical and neuroimaging data of a large genetically defined cohort of 47 patients with *FLNA*-associated PVNH. Based on the combined data of previous reports and our results we emphasize the importance of early cerebral imaging in females with a history of patent ductus arteriosus and seizures, regardless of their age of onset or cognitive impairment.

Identification of the underlying *FLNA* mutation should prompt inclusion in regular intensified medical care in an epilepsy center as well as cardiovascular surveillance by physicians familiar with the wide phenotypic spectrum of *FLNA* mutation carriers. Asymptomatic *FLNA* mutation carriers may have epileptic discharges, but there is currently no evidence, that they may benefit from antiepileptic medication. *FLNA*-associated seizures may occur at any age, onset during adolescence or in adulthood is not uncommon. Antiepileptic medication is effective in reducing seizure frequency, but about one third of the patients may not be free of seizures even with multimodal medication protocols. Whether or not lamotrigine alone or in combination with specific add-on compounds will turn out to be most effective to control *FLNA*-associated seizures, is currently addressed by our group in an ongoing clinical study.

Our data on this largest currently published cohort of *FLNA* mutation carriers with characteristic PVNH on MR imaging confirm a preferentially normal cognitive development or only mild impairment, which should not prevent further genetic workup. A clear genotype-phenotype correlation was not obvious in our series. However, early seizure onset in childhood may more likely be associated with additional cardiovascular, gastrointestinal and musculoskeletal manifestations. Earlier genetic diagnosis of this X-linked dominant inherited disorder during the process of clinical workup of PVNH identified in neuroimaging should be sought in order to offer non-directive genetic counseling prior to childbearing to affected females as well as their close relatives. Genetic diagnosis should also warrant a thorough, systematic, general medical work-up to identify common and less frequently observed clinical findings affecting connective tissue, lung as well as cardiovascular or gastrointestinal function and to organize a specialized interdisciplinary medical care and surveillance to ultimately further improve long term outcome and quality of life of the affected patients and their families.
